# Supplementary material for: Transcriptome Analysis on the Mechanism of Ethylicin Inhibiting Pseudomonas syringae pv. actinidiae on Kiwifruit
Source: Microorganisms. 2021 Mar 31;9(4):724. doi: 10.3390/microorganisms9040724 (PMC8067213; doi:10.3390/microorganisms9040724)
Supplement: Supplementary file 1 [file microorganisms-09-00724-s001.zip › supporting inforamtion/figure for supplement.docx]

Article

Transcriptome analysis on the mechanism of ethylicin inhibiting *Pseudomonas syringae* pv. *actinidiae* on Kiwifruit

Tao Liu ^1^, Xiaoli Ren ^1^, Guangyun Cao ^1^, Xia Zhou ^1,^* and Linhong Jin ^1,^*

^1^ State Key Laboratory Breeding Base of Green Pesticide and Agricultural Bioengineering, Key Laboratory of Green Pesticide and Agricultural Bioengineering, Ministry of Education, Guizhou University, Huaxi District, Guiyang 550025, China; gs.taoliu18@gzu.edu.cn (T.L.); gs.xlren20@gzu.edu.cn (X. R.); gs.gycao20@gzu.edu.cn (G. C.)

* Correspondence: lhjin@gzu.edu.cn (L. J.); xzhou@gzu.edu.cn (X. Z.). Tel.: +86-851-3620-521(L.J. & X.Z.)


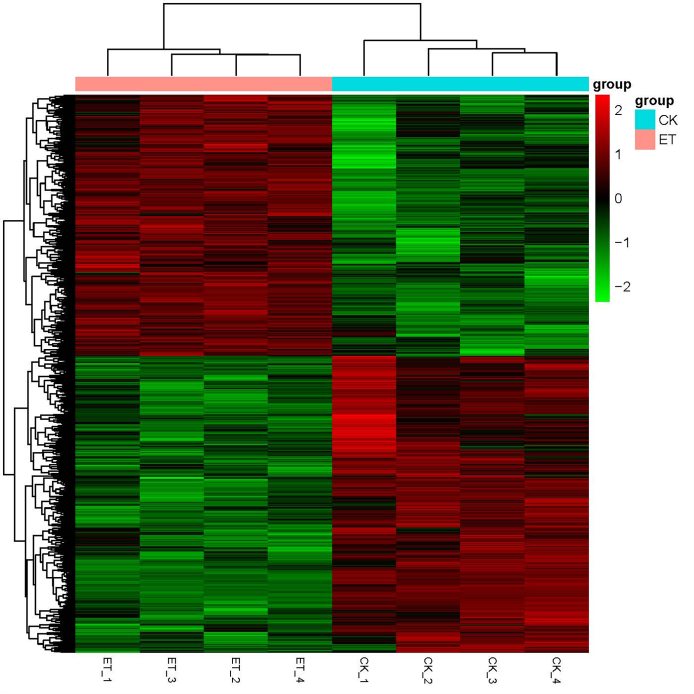


Figure S1: Differential gene clustering heat map.


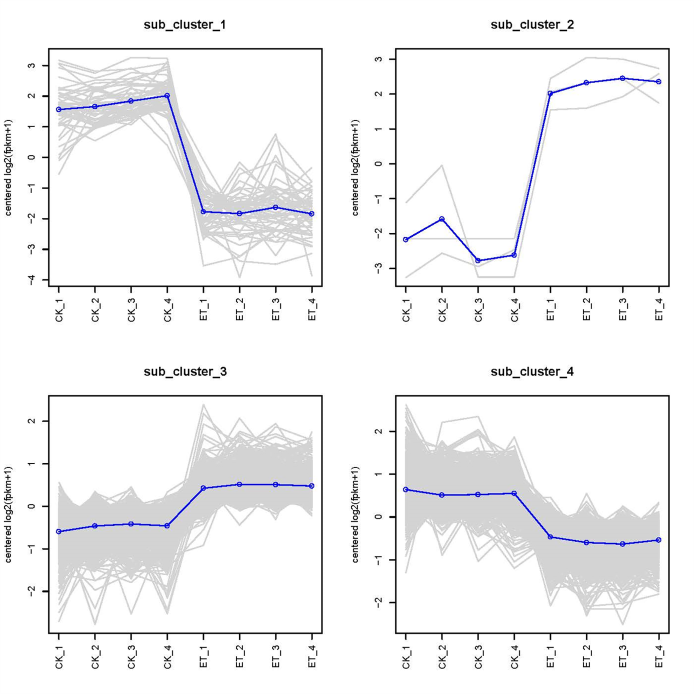


Figure S2: Differential gene clustering line chart.


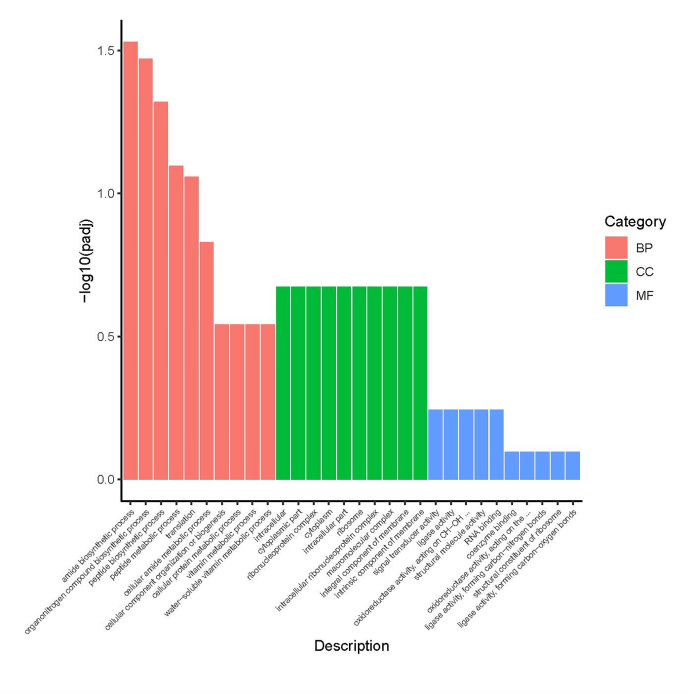


Figure S3: GO enrichment analysis.


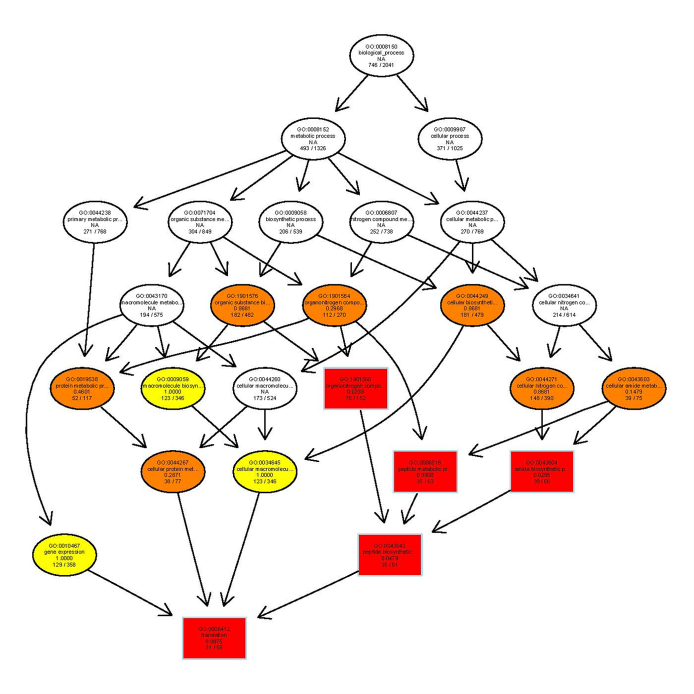


Figure S4: BP Directed Acyclic Graph.


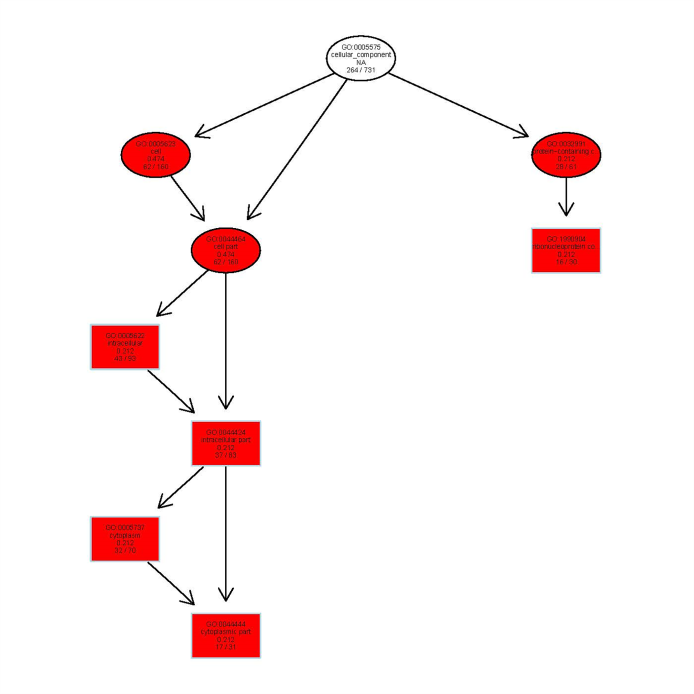


Figure S5: CC Directed Acyclic Graph.


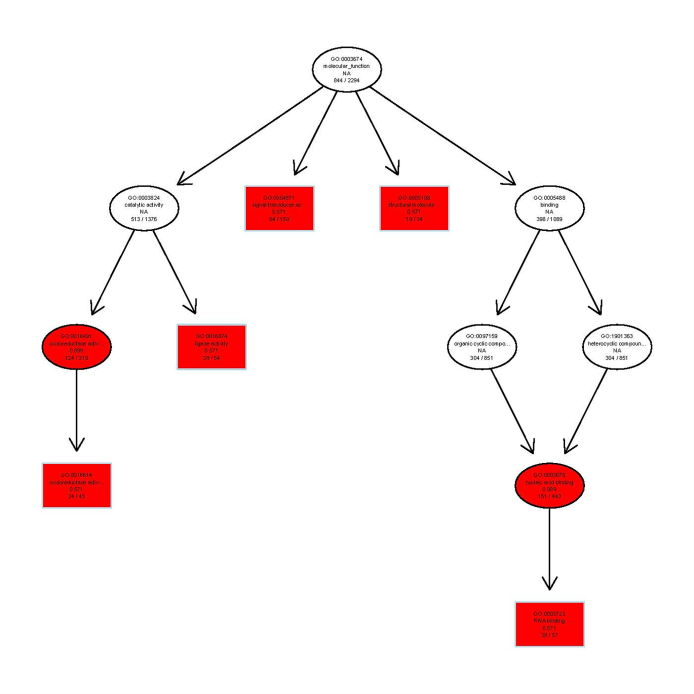


Figure S6: MF Directed Acyclic Graph.


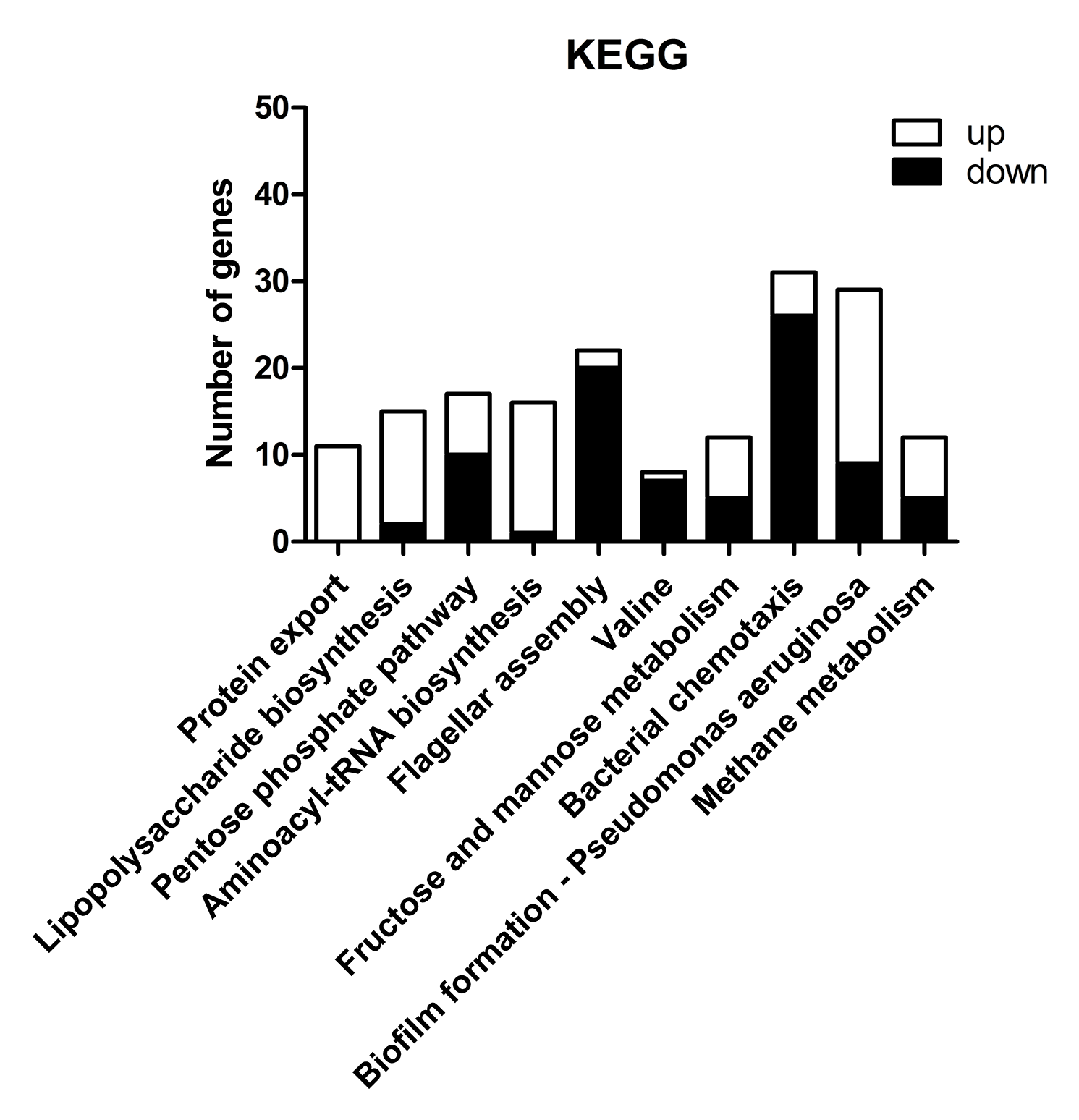


Figure S7: Statistics of the number of differential genes in the top 10 pathways enriched by KEGG.


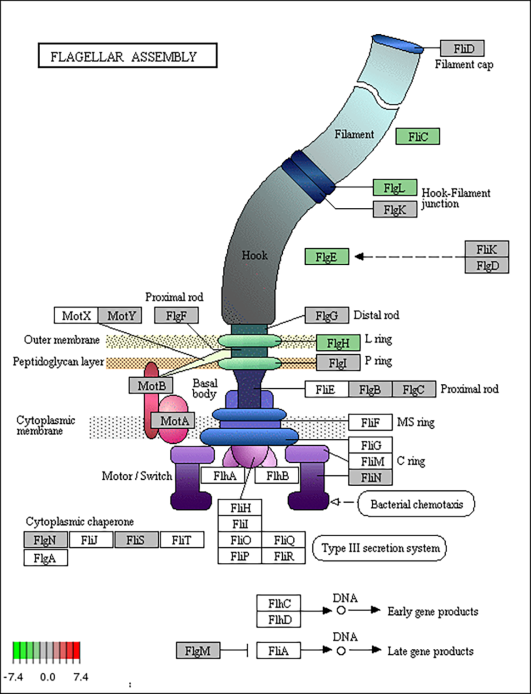


Figure S8: Flagellar assembly pathway.


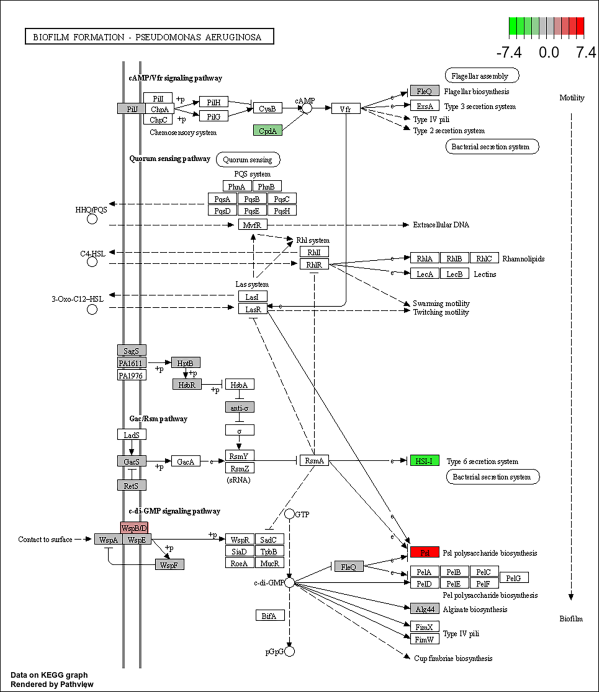


Figure S9: Biofilm formation-Pseudomonas aeruginosa pathway.
